# A use/disuse paradigm for CRISPR-Cas systems

**DOI:** 10.1007/s10539-018-9661-z

**Published:** 2019-02-11

**Authors:** Sophie Juliane Veigl

**Affiliations:** University of Vienna, Vienna, Austria

In his insightful review, Eugene V. Koonin discusses various aspects of CRISPR-Cas systems with a strong focus on their qualities as “adaptive (acquired) immune systems” (Koonin 2018, 3). The CRISPR-Cas system is most famous for its application as a gene-editing tool. Koonin provides a deeper insight into its biological function in bacteria, which is to immunize the cell against parasite DNA. I shall comment on one issue discussed in the text, in two steps. First, I shall elaborate on CRISPR-Cas systems and their supposed Lamarckian character. Criteria for calling biological phenomena genuinely Lamarckian will be narrowed down and then applied to the CRISPR-Cas system, considering interference-driven spacer acquisition (IDSA) as an instantiation of a truly Lamarckian paradigm. Second, I shall consider whether Lamarckian and “canonical” instances of inheritance are a case of theoretical pluralism, being two non-reducible, yet interconnected paradigms.

Koonin suggests that CRISPR-Cas based inheritance of acquired traits (IAC) might be an example for a genuine Lamarckian mechanism, because it operates by “(1) specific, heritable changes in the genome caused by an external factor, (2) specific phenotypic effect of those changes that constitutes adaptation to the causative factor” (Koonin 2018, 6f). Yet, this definition is not exclusively referring to Lamarck’s musings. For example, such properties are also associated with Darwin’s “gemmuli” or “pangenesis” [which have recently been quite more beloved metaphors than any of Lamarck’s concepts (Chen et al. 2016)]. Nevertheless, it is possible to differentiate the general idea of IAC from a genuinely Lamarckian theme. Lamarck proposed a mechanistic paradigm that coordinates the inheritance of acquired traits: the “use/disuse paradigm.” This paradigm postulates that through continuous “use” of certain “organs” these “organs” will be augmented. Contrarily, through “disuse” of certain “organs”, these “organs” will be reduced (Lamarck 1809 cited in Burkhardt 1995). Naturally, we need to grant some interpretative charity when working with the terms “organ” and “use/disuse”. Notably, working with Lamarck’s use/disuse paradigm is of great epistemological value for understanding other instances of IAC (Veigl 2017).

I want to propose a way how one could fit CRISPR-Cas into a use/disuse paradigm. In so doing, we must shift our attention to one distinct feature of use/disuse, namely its focus on the quantitative aspects of the process. In a use/disuse scenario, we do not primarily deal with qualitative yes/no phenomena, but with gradual processes. Therefore, we shall consider pools of crRNA species instead of discrete spacers in CRISPR arrays. This should also help to understand the continuum between Darwinian and Lamarckian types of inheritance (Koonin 2018, 13), as they might work upon different substrates (DNA vs. RNA), integrating differently from case to case. One instance of the use/disuse paradigm is interference-driven spacer acquisition (IDSA) (Staals et al. 2016; Hille et al. 2018). IDSA links interference to adaption, meaning that crRNA/CRISPR targeted parasite DNA becomes a source for new spacers which are integrated into the CRISPR array. The newly acquired spacers are slightly different in sequence but target the same parasite DNA.

With IDSA and the use/disuse paradigm at hand I shall now analyze the phase of interference. Here, we have to consider that Cas molecules as well as their targets (nucleic acids) are present in different concentrations. There is a pool of Cas proteins, and there is a pool of crRNAs. As a result of differences in sequence, each crRNA deriving from a spacer region has slightly different biochemical properties. Such differences might facilitate or impede binding. Ensuing expression, a pool of various crRNAs competes for binding to Cas molecules. What we shall define as “use” is a situation in which a specific crRNA (crRNA 1) binds a Cas protein and targets a parasite DNA sequence (thus both, binding to Cas and the parasite DNA, are required for “use”). Protospacers will be salvaged and inserted into the CRISPR array during elimination of the parasite nucleic acid. In the next round of expression, there will be a higher concentration of crRNA 1-associated crRNAs, and this subspecies is more likely to bind to a Cas protein again, as a result of its higher abundance. Thus, we have augmentation through use. Of course, use and disuse go hand in hand, as more use of one subspecies implies disuse of other species and their relative reduction in abundance. It has been shown that CRISPR-Cas complexes show a preference for more recently acquired spacers, cementing disuse and reduction on the RNA level for less used spacers. In addition, there are optimal numbers for spacers in CRISPR arrays (Koonin 2018, p. 15; Martynov et al. 2017) and thus, spacers must be lost over time.

The case of IDSA shows that shifting our attention to other phases than the DNA phase of CRISPR will help us to find resonance with a modern interpretation of Lamarck’s “use/disuse paradigm.” DNA might be the genuine playground of Darwinian paradigms, yet this does not mean that other material sources are not present. It is often claimed that all forms of inheritance are in the end reducible to DNA-based mechanisms, as the relevant biomolecules, such as the crRNAs, derive from it. Yet one must not confuse DNA’s primacy in biochemistry with that in inheritance and evolution, as here, clearly, other levels can play a role, as shows use/disuse of crRNAs.

I want to highlight one possible implication of CRISPR-Cas for philosophical problems, namely its potential for discussions centering around scientific pluralism. I define scientific pluralism here very broadly as the claim that to approach a certain phenomenon, several non-reducible theories, explanations or methods are, might be or should be required (see Kellert et al. 2006). For the case considered, we shall adopt the pluralist attitude that there are (at least) two non-reducible mechanistic paradigms that govern the processes of inheritance. These two mechanistic paradigms are based on two different theoretical approaches: IAC versus Natural Selection through Random Variation. In addition, both approaches have a different understanding of the interplay between the (inducing as well as selecting) environment, inheritance and evolution. We will add, to be fully-fledged pluralists, that we are approving of this situation, because we find it either epistemologically beneficial or claim that this kind of plurality is metaphysically resonant with what the world is like. (I, personally, refrain from the latter and find the former sufficiently desirable.) Now we shall turn again to the phenomenon at stake: CRISPR-Cas systems produce types of inheritance (that might have evolutionary impact) which are not included in an up-to-date version of the modern synthesis. Although CRISPR arrays are subjected to Darwinian random mutation, the arrays also adapt through a Lamarckian type, directed adaption, orchestrated by a use/disuse paradigm. Of course, both processes do not exclude each other, in principle. Yet, it is one key feature of the modern synthesis’ supporters to exclude any instances of IAC, especially forms that can be associated with Lamarck’s theory.

Now, how shall we characterize this instance of pluralism? What kind of attack on orthodoxy does it pose if one shows that some core theoretical assumptions do not universally apply? Let me draw an analogy from a core theoretical assumption in physics: the first law of thermodynamics. Is claiming “sometimes Lamarckian inheritance is instantiated” the same as claiming “sometimes energy is not conserved”? What is now key here is that the answer might be “yes” and “no”. To some, such an analogy is valid (Haig 2007). I would call these researchers “Neo-Darwinian singularists.” Yet, to others the answer is clearly “no”: Scientists insisting on instances of Lamarckian inheritance usually accept all instances where a Darwinian framework has more explanatory power and also accept “compound cases”, where integration of both modes explains a phenotype (Koonin 2018, 13). I suggest calling these actors “dualists”. I am not conscious of actors who would fit the definition of “Lamarckian singularists”, insisting that all instances of inheritance and evolution are strictly Lamarckian. Thus, we are encountering certain tensions and assymetries, both for the perspective of the philosopher versus actors in the research field, as well as within the scientific community. Whereas “pluralism” seems to be a category mainly used by philosophers, the categories of “dualism” and “singularism” might better describe the positions researchers take. Individual takes of researchers concerning pluralism are not defined by them being dualist or singularist. Yet one could suspect that dualism might invite a pluralist interpretation of the phenomenon at stake. To conclude, CRISPR-Cas teaches us a lot about alternative trajectories of inheritance, which might constitute an assymmetric case for theoretical pluralism. Nevertheless, we should pay close attention to the actors within the respective field, who will finally negotiate the rise and decline of plurality in their research area.

## References

[CR1] Burkhardt RW (1995). The spirit of system: Lamarck and evolutionary biology: now with “Lamarck in 1995”.

[CR2] Chen Q, Yan W, Duan E (2016). Epigenetic inheritance of acquired traits through sperm RNAs and sperm RNA modifications. Nat Rev Genet.

[CR3] Haig D (2007). Weismann rules! OK? epigenetics and the Lamarckian temptation. Biol Philos.

[CR4] Hille F (2018). The biology of CRISPR-Cas: backward and forward. Cell.

[CR5] Kellert SH, Longino HE, Waters CK (2006). Scientific pluralism.

[CR9] Koonin EV (2018). CRISPR: a new principle of genome engineering linked to conceptual shifts in evolutionary biology. Biol Philos.

[CR6] Martynov A, Severinov K, Ispolatov I (2017). Optimal number of spacers in CRISPR arrays. PLoS Comput Biol.

[CR7] Staals RHJ (2016). Interference-driven spacer acquisition is dominant over naive and primed adaptation in a native CRISPR-Cas system. Nat Commun.

[CR8] Veigl SJ (2017). Use/disuse paradigms are ubiquitous concepts in characterizing the process of inheritance. RNA Biol.

